# Glutamatergic neurometabolite levels in the caudate are associated with the ability of rhythm production

**DOI:** 10.3389/fnins.2023.1196805

**Published:** 2023-08-04

**Authors:** Shiori Honda, Yoshihiro Noda, Karin Matsushita, Ryosuke Tarumi, Natsumi Nomiyama, Sakiko Tsugawa, Yui Tobari, Nobuaki Hondo, Keisuke Saito, Masaru Mimura, Shinya Fujii, Shinichiro Nakajima

**Affiliations:** ^1^Department of Neuropsychiatry, Keio University School of Medicine, Tokyo, Japan; ^2^Seikeikai Komagino Hospital, Hachioji, Japan; ^3^Faculty of Environment and Information Studies, Keio University, Kanagawa, Japan; ^4^Multimodal Imaging Group, Research Imaging Centre, Centre for Addiction and Mental Health, Toronto, ON, Canada

**Keywords:** magnetic resonance spectroscopy, rhythm, glutamate, caudate, rhythm production

## Abstract

**Introduction:**

Glutamatergic neurometabolites play important roles in the basal ganglia, a hub of the brain networks involved in musical rhythm processing. We aimed to investigate the relationship between rhythm processing abilities and glutamatergic neurometabolites in the caudate.

**Methods:**

We aquired Glutamatergic function in healthy individuals employing proton magnetic resonance spectroscopy. We targeted the right caudate and the dorsal anterior cingulate cortex (dACC) as a control region. Rhythm processing ability was assessed by the Harvard Beat Assessment Test (H-BAT).

**Results:**

We found negative correlations between the production part of the Beat Saliency Test in the H-BAT and glutamate and glutamine levels in the caudate (*r* = −0.693, *p* = 0.002) whereas there was no such association in the dACC.

**Conclusion:**

These results suggest that higher glutamatergic neurometabolite levels in the caudate may contribute to rhythm processing, especially the ability to produce meter in music precisely.

## 1. Introduction

Music contains rhythm, which configures patterns of time intervals. Previous studies noted that dopamine plays an important role in auditory rhythm processing (Grahn, 2009; Koshimori et al., 2019). According to a neuropharmacological study, the glutamatergic system may be involved in time perception by interacting with the dopaminergic system (Cheng et al., 2007). For example, an animal study reported that inhibiting glutamatergic function enhanced dopaminergic function, resulting in altered time perception (Cheng et al., 2007). These findings suggest that glutamatergic function may be related to music rhythm processing.

However, few animal studies reported the relationship between music and glutamatergic function. One study showed that exposing musical stimuli induced the expression of the glutamatergic AMPA receptor in mice (Xu et al., 2007). In addition, listening to music during childhood induced the expression of the glutamatergic NMDA receptor subunit NR2B protein in the auditory cortex, which enhanced the development of auditory functions (Xu et al., 2009). Another study reported that glutamatergic neurometabolite concentrations in the striatum were decreased with sad music called “Shange,” which is one of the Chinese traditional music therapy, and joyful and powerful music called “Zhi” and “Gong” increased its concentrations (Hao et al., 2020). However, these previous studies have the following limitations: (1) they were performed for only rodent models, and (2) they used musical stimuli which include changes not only in rhythm but also in melody, harmony, and timbre to assess the relationship between music and glutamatergic function. Therefore, the relationship between musical rhythm processing and glutamatergic neuro-systems remains unclear.

The striatum has been shown to be closely linked to the perception and production of musical rhythms (Grahn, 2009; Grahn and Rowe, 2013). Previous reports have demonstrated that the striato-thalamo-cortical network is particularly activated when processing beat-based rhythms in music (Grahn, 2009; Grahn and Brett, 2009; Teki et al., 2011a,b). Grahn and Rowe et al. have established that activity in the basal ganglia increases during the processing of musical rhythms (Grahn and Brett, 2009), and patients with Parkinson’s disease who have dopamine dysfunction exhibit impairments in their rhythm perception (Grahn and Rowe, 2009). Additionally, the striatum has been identified as a central region where the dopamine and excitatory-inhibitory systems (glutamate – gamma-aminobutyric acid functions) interact (Agnoli et al., 2013).

Based on these findings, we hypothesized that glutamate levels in the striatum may be related to musical rhythm perception and production. Hence, the present study sought to investigate whether glutamatergic neurometabolite levels in the striatum relate to the rhythm processing ability in humans.

In this study, we quantified the concentrations of glutamatergic neurometabolites in the caudate as a region of interest employing proton magnetic resonance spectroscopy (^1^H-MRS). As a control region, the dorsal anterior cingulate cortex (dACC) was selected from our previous study in an exploratory fashion (Tarumi et al., 2020). Tarumi et al. (2020) compared Glx levels in the caudate and dACC among patients with treatment-resistant schizophrenia, patients with treatment-responsive schizophrenia, and healthy controls. For the present study, we analyzed the data acquired from the same healthy subjects as in Tarumi et al. (2020). Given that the ACC plays an important role in global executive function, we hypothesized that we could not discern music-specific functions from this region. Thus, we set the dACC as a positive control ROI.

## 2. Methods

### 2.1. Participants

The study was approved by the ethics committees at Komagino Hospital, Keio University School of Medicine, and Keio University Shonan Fujisawa Campus. All methods were carried out in accordance with the relevant guidelines and regulations expressed in the Declaration of Helsinki. All participants provided written informed consent prior to enrollment. Thirty-three healthy individuals participated in this study via a private committee for recruitment (Table 1). All participants were screened by qualified psychiatrists (R.T., Y. N, and S.N.) based on the Diagnostic and Statistical Manual of Mental Disorders, Fifth Edition (DSM-5). The exclusion criteria of participants were a history of psychiatric disorders, neurological, or significant medical disorders. All experiments were performed at Komagino Hospital. All MRI images were shared with Tarumi et al. (2020).

**Table 1:** Demographic information.

Measures (mean ± SD)		
Number of participants		33
Number of participants for analyses	Caudate	22
	dACC	27
Age, years		43.233±11.849
Number of females		13
Duration of music training, years		5.032±7.468
Averages of H-BAT measures		
Beat Interval Test (BIT), log_2_ms	Perception	–1.568±1.501
	Production	–2.784±1.218
Beat Finding and Interval Test (BFIT), log_2_ms	Perception	–1.232±1.377
	Production	–2.029±1.903
Beat Saliency Test (BST), log_2_dB	Perception	0.823±1.128
	Production	1.615±1.488

The number of participants, Mean and standard deviation (SD) of age, number of females, and duration of musical training in years are shown.

### 2.2. Magnetic resonance imaging

All images were acquired by a 3T GE Signa HDxt scanner with an eight-channel head coil. We assessed a three-dimensional inversion recovery prepared T1-weighted magnetic resonance imaging (MRI) scan (Axial MRI 3D brain volume (BRAVO), echo time (TE) = 2.8, repetition time (TR) = 6.4, inversion time (TI) = 650 ms, flip angle = 8°, field of view (FOV) = 230 mm, 256 × 256 matrix, slice thickness = 0.9 mm). MR scanning as described in Tarumi et al. (2020).

### 2.3. Acquisition of glutamatergic levels and data processing

**Figure 1:**
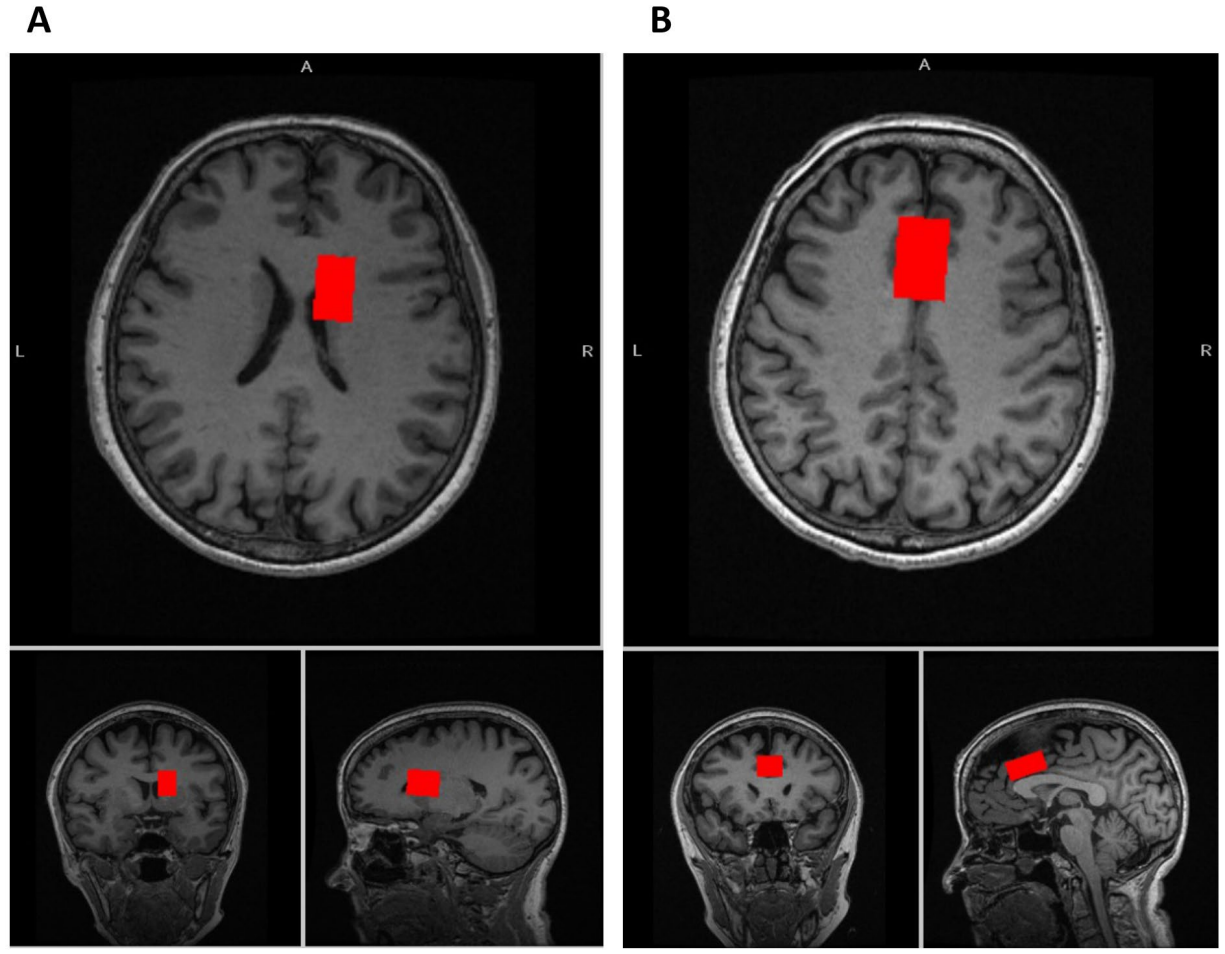
Voxel locations of MRS. **(A)** The voxel location of the caudate (voxel size: 7.5 mL [2.5 × 1.5 × 2.0 cm^3^]). **(B)** The voxel location of the dorsal anterior cingulate cortex (dACC) (voxel size: 9.0 mL [3.0 × 2.0 × 1.5 cm^3^]).

**Figure 2:**
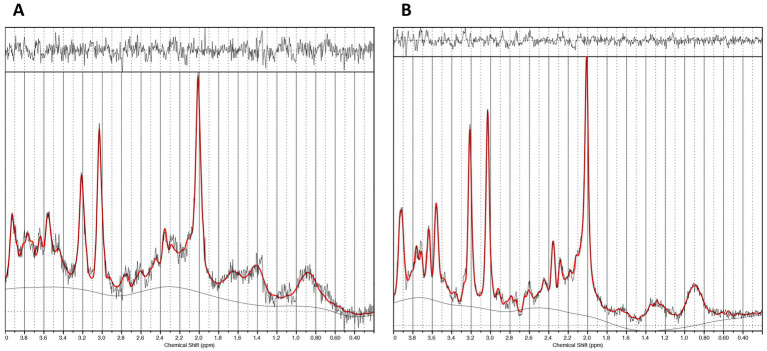
Representative spectra. **(A)** The caudate ^1^H-MRS spectra. **(B)** The dACC ^1^H-MRS spectra.

We acquired glutamatergic neurometabolite levels using ^1^H-MRS. The scanning parameters were as follows: PRESS, TE = 35 ms, TR = 2000 ms, spectral width = 5000 Hz, 4096 data points, 128 water-suppressed, 16 water-unsuppressed averages, and 8 numbers of excitation. The locations of the ^1^H-MRS voxels, and representative spectra are provided in Figures 1, 2. The voxels were placed on the right caudate (voxel size = 7.5 mL) and bilateral dACC (voxel size = 9.0 mL), based on the aims of another project (Tarumi et al., 2020). In this study, we used Glx levels, a combination of glutamate and glutamine. It is because the molecular structures and molecular weights of Glu and Gln are similar, and the spectrum peaks overlap, making it difficult to discriminate between them using 3T MRI. We employed the FID-Appliance for pre-processing of spectra, primarily for estimation and correction of frequency and phase drifts^1^ (Simpson et al., 2017). Subsequently, we estimated neurometabolite levels utilizing a basis set, and extracted values that were normalized to the unsuppressed water signal from LCModel outputs with institutional units. The authors visually inspected all spectra exported from LCModel. Furthermore, we established criteria for spectra quality and excluded spectra that failed to meet the following criteria: signal-to-noise ratios (SNR) ≤10, full-width at half maximum (FWHM) ≥10 Hz, or %SD values ≥20%. To correct for voxel tissue composition, we segmented the T1-weighted image into gray matter (GM), white matter (WM), and cerebrospinal fluid (CSF) using FSL (FMRIB Software Library v5.0, Oxford, UK). Subsequently, we generated individual masks that contained information about voxel size and location on the segmented T1-weighted images using GANNET.^2^ To acquire the observed metabolite concentrations with respect to a relatively and fully relaxed water peak from tissue [M], we took into account the effects of volume fractions, water relaxation times (T1, T2), and water concentrations for the three compartments (WM, GM, and CSF). We performed calculations that considered LCModel operations as follows:


$$\begin{array}{l} \left[\mathrm{M]}=[{\mathrm{M]WS}}^{*}[({\mathrm{fCSF}}^{*}55556^{*}\mathrm{RCSF})+({\mathrm{fGM}}^{*}43300^{*}\mathrm{RGM}\right)\\ +\;({\mathrm{fWM}}^{*}35880^{*}\mathrm{RWM})]/({\mathrm{FLC}}^{*}(1-\mathrm{fCSF})) \end{array}$$

Where [M] WS is water-scaled data from LCModel. And FLC is an LCModel factor that is used to undo the assumptions used by LCModel [i.e., FLC = WCONC*ATT20; WCONC = 35880 and ATT20 = 0.7 = exp(−30/80)].


RT=(1−exp(−TR./T1T)∗exp(−TE./T2T))

where, fT and RT are the volume fraction and water relaxation parameters of tissue T (T = GM, WM, and CSF of the voxel), respectively. Relaxation times and relative water tissue content values are outlined in Supplementary Table 1. And, spectrum qualities and tissue heterogeneity values are shown in Supplementary Table 2.

### 2.4. Assessments for rhythm perception and production abilities

Rhythm perception and production abilities were assessed with the Harvard Beat Assessment Test (H-BAT) (16). The H-BAT consists of three subtests. (1) Beat Interval Test (BIT) in which the participants were discriminated if the tempo of a metronome was getting faster or slower (BIT perception), then tap in synchrony with the tempo-changing metronome without discrimination of temporal changes (BIT production). (2) Beat Finding and Interval Test (BFIT) in which the participants discriminated if the tempo of a rhythm pattern was getting faster or slower (BFIT perception), then tap the quarter-note beat with the tempo-changing rhythm pattern without discrimination of temporal changes (BFIT production). (3) Beat Saliency Test (BST) in which the participants discriminated if a sequence of accented quarter-notes was a duple or triple meter (BST perception), then produce the meter by changing the tap amplitudes without discrimination which meter they heard (BST production). In brief, BIT and BFIT assess the sensitivity to temporal change in non-isochronous tone sequences while BST assesses the sensitivity to amplitude change in isochronous tone sequences (Fujii and Schlaug, 2013). Each of the perception subtests assess the sensory process while that of production subtests assess the sensorimotor process.

The performance of BIT, BFIT, and BST in the H-BAT was quantified with perception and production thresholds. The lower the thresholds, the more precisely the participant perceives and produces the rhythms. The thresholds were normalized by log transformation with the base of two based on the previous study (Fujii and Schlaug, 2013; Paquette et al., 2017). For more details about the tests and analyses on the H-BAT, see the previous studies (Fujii and Schlaug, 2013; Paquette et al., 2017).

### 2.5. Statistical analysis

Statistical analyses were carried out using IBM SPSS Statistics version 26 (IBM Corporation, Armonk, NY). To account for the effect of music training, we calculated the standard division (SD) of the duration of music training for all participants. If the duration of music training exceeded ±2SD, the participant was excluded as an outlier from subsequent analyses. First, we performed partial correlation analyses by Pearson’s method to examine the relationship between the H-BAT measures and glutamatergic levels in dACC and caudate using age and sex as covariates. Second, partial correlation analyses were performed to examine the effect of the duration of music training. All results of partial correlation analyses are also adjusted by the Bonferroni method. The significance level was *p* < 0.004 (*p* < 0.05/n where n equals the number of ROIs and tests).

## 3. Results

Demographic information is shown in Table 1. Sixteen individuals have musical training imparted by professionals, excluding education in mandatory school. The breakdown of instruments is as follows: piano, 12; organ, 1; flute, 1; saxophone, 1; erhu, 1. A total of 22 and 27 participants’ data were used for the analyses of the caudate and dACC, respectively. At the time of acquisition, we excluded 2 HCs who did not complete scans and 2 HCs with incidental brain anomalies. Further, the data of 2 participants were missing because of a technical issue with the H-BAT application. Regarding statistical analyses, 4 participants’ data on the caudate were excluded due to low SNR values, and 1 participant was rejected through the preprocessing for the spectrum. If the duration of music training exceeded ±2SD, the participant was excluded as an outlier from subsequent analyses (see Supplementary Figure 1).

**Figure 3:**
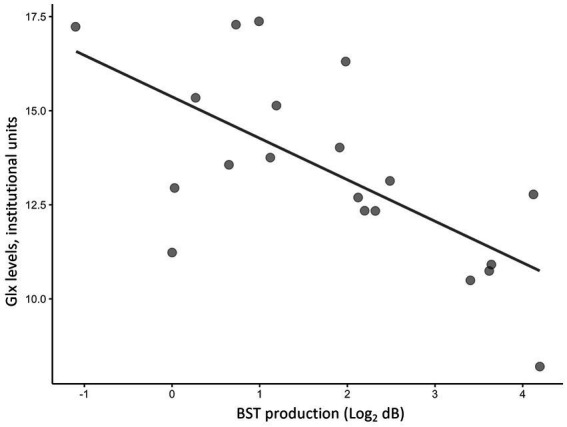
A scatter plot of a correlation between Glx levels in the caudate and BST production scores.

Partial correlation analyses using age and sex as covariates showed significant correlations between H-BAT subscores and Glx levels in the caudate or the dACC. Table 2 shows the correlation between the H-BAT measures and Glx levels in the caudate and dACC using age, sex, and the duration of musical training. There was a significant correlation between the BST production threshold and Glx levels in the caudate (Figure 3), while no association was found in the other H-BAT measures. We conducted the correlation analyses including the outlier data as a sensitivity analysis. We still had a significant correlation between BST perception and Glx levels in the caudate. On the other hand, in the ACC, no significant relationship was found between Glx levels and any of the H-BAT measures.

**Table 2:** Results of correlation between H-BAT scores and Glx levels in the caudate and the dACC.

			Caudate	dACC
Beat Interval Test	Perception	Coefficient	−0.285	−0.003
		*p* value	0.268	0.989
	Production	Coefficient	−0.533	0.238
		*p* value	0.028	0.299
Beat Finding and Interval Test	Perception	Coefficient	−0.556	0.121
		*p* value	0.020	0.602
	Production	Coefficient	0.231	0.465
		*p* value	0.373	0.034
Beat Saliency Test	Perception	Coefficient	0.157	0.055
		*p* value	0.548	0.814
	Production	Coefficient	−0.693	0.301
		*p* value	0.002*	0.184

* significant correlations (Bonferroni corrected, *p* < 0.004).

## 4. Discussion

This is the first ^1^H-MRS study to examine the relationship between rhythm perception and production abilities measured with the H-BAT and glutamatergic levels in the caudate of healthy individuals. We found a negative relationship between BST production thresholds and Glx levels in the caudate in healthy individuals. On the other hand, no association was detected between the other H-BAT measures and Glx levels in the caudate, or between any H-BAT measures and Glx levels in the dACC (a control region). These results suggest that higher Glx levels in the caudate may specifically reflect the ability to produce a more precise isochronous meter.

What is the role of glutamatergic function in time processing? The dopaminergic function in the striatum has been shown to play an important role in time processing while the glutamatergic function in time processing remains unclear. Cheng et al. (2007) performed a pharmacological study in rats using cocaine, a dopamine transporter blocker, and ketamine, a glutamate receptor antagonist. They showed that cocaine disrupted time perception and ketamine augmented the time disruption modulated by cocaine. This animal study suggests that the dopamine and glutamate pathways may interact with each other to process time (Cheng et al., 2007). In humans, it was noted that time perception was distorted in patients with schizophrenia where the dopamine and glutamate systems are impaired (Carroll et al., 2008). The dopamine dysfunction in the dorsal striatum, one of the pathological hypotheses for schizophrenia, may be caused by glutamatergic dysfunction in patients with schizophrenia (Flagstad et al., 2004; Wada et al., 2022). This study adds evidence to support the relationship between striatal glutamate levels and rhythm processing mechanisms in humans *in vivo*.

Why was the correlation found only in BST production but not in the other H-BAT measures? This correlation may be attributed to the specific characteristics of BST stimuli. Unlike BIT and BFIT, which use non-isochronous time intervals in their stimuli, BST uses isochronous time intervals. Specifically, the sound stimulus in BST consisted of a tone sequence of 500-msec isochronous time intervals with accented and unaccented tones (Fujii and Schlaug, 2013). To perform BST, participants had to encode the relative-intensity difference between the accented and unaccented tones precisely overtime to process the duple or triple meter precisely. Namely, it is crucial to encode the meter or an organization of sound intensity over time in isochronous intervals in BST. On the other hand, BIT and BFIT use non-isochronous time intervals without any accents. Both BIT and BFIT include gradual changes in time intervals to create a faster or slower tempo (Fujii and Schlaug, 2013). In BIT and BFIT, each interval in the stimuli is different, and therefore, encoding of the absolute duration of time intervals is considered to be important. A previous study noted that there was a difference in neural circuits in the brain when we process absolute and relative time intervals (Grube et al., 2010a,b; Teki et al., 2011a,b). The absolute, duration-based time intervals are considered to be processed in the olivocerebellar network, while the relative, beat-based time intervals are considered to be processed in the striato-thalamo-cortical network (Teki et al., 2011a,b). Considering the results of this study and these separated mechanisms of rhythm processing in the brain, we assume that BIT and BFIT may assess relatively olivocerebellar-based rhythm ability, while BST may assess striato-thalamo-cortical-based rhythm ability. In fact, our previous study showed that the gray-matter volume in the cerebellum was correlated with the BIT and BFIT scores but not with the BST score in the H-BAT in healthy individuals (Paquette et al., 2017). Therefore, these findings suggest that glutamatergic neurometabolite levels in the striatum may contribute to the processing of meter or temporal organization in isochronous time intervals.

Why does this effect appear in the production test but not in the perception test? This discrepancy may be attributed to the role of the striatum in motor output and auditory-motor interaction. Mounting evidence suggests that the cortico-striatal network has an important role in encoding and retrieving motor information; and also see a review by Miyachi et al. (1997), Matsumoto (1999), and Kotz et al. (2009). To perform BST production, participants are required to encode the pattern of accented and unaccented tones precisely as well as produce the meter as motor output by modulating their tapping amplitudes. Conversely, the perception test does not require the same level of motor output, such as the physical articulation of rhythm sequences. Hence, our results suggest that Glx levels in the caudate contribute to the encoding of auditory meter information, the auditory-motor transformation of the meter, and the significant role of motor output. On the other hand, in light of the statistical power of this study, we may not rule out the potential of other rhythm components which relate to glutamatergic function in the caudate. We need to consider differences in the relationship between various types of rhythm components and glutamatergic function in future studies.

We did not find any significant relationship between the H-BAT measures and glutamatergic neurometabolite levels in the dACC. Previous studies reported that both regions play important roles in cognitive monitoring, motor control, and association of perception-production (Bush et al., 2002; Maes et al., 2014; Brockett et al., 2020) and there are structural and functional connectivities between the dACC and striatum (Beckmann et al., 2009). However, our findings suggest that Glx levels in the striatum are more directly related to rhythm or meter processing compared to those in the dACC. Further research is needed to examine the interaction between glutamatergic functions in the dACC and caudate and its relationship to rhythm processing.

There are several limitations to this study. Firstly, our acquisition was solely based on a resting-state quantitative ^1^H MRS, averaged over time rather than functional MRS employing beat processing tasks Secondly, our research did not measure the voxel in another basal ganglia region. Previous reports have suggested distinct roles of the putamen and caudate in rhythm processing (Coull et al., 2011; Grahn and Rowe, 2013). Consequently, our study was unable to determine whether glutamate levels in each striatal subregion are different or the same in their relation to rhythm processing. Thirdly, we were unable to discern the precise origin of the glutamatergic signal, i.e., whether it was inside or outside the cells. The limitation of MRS only allowed for identifying an averaged glutamatergic signal from all receptors within the placed voxel, given the absence of pharmacological tracers.

## 5. Conclusion

In conclusion, we found that glutamatergic neurometabolite levels in the caudate were associated with the ability to produce rhythm or meter in healthy individuals. This result suggests that the neurometabilite levels measured with ^1^H-MRS contribute to further understanding of musical rhythm processing. We propose that a multimodal measurement approach would be efficacious in furthering our understanding of the neurometabolite mechanisms underlying musical rhythm processing in humans.

## Data availability statement

The raw data supporting the conclusions of this article will be made available by the authors, without undue reservation.

## Ethics statement

The studies involving human participants were reviewed and approved by the Ethics Committees at Komagino Hospital, Keio University School of Medicine, and Keio University Shonan Fujisawa Campus. The patients/participants provided their written informed consent to participate in this study.

## Author contributions

SH recruited healthy participants, collected the data, analyzed the dataset, and wrote the manuscript. YN, SF, and SN contributed to the study design, wrote the manuscript, and supervised this study. KM and NN collected the data and contributed to writing the manuscript. RT recruited patients and collected the data. ST analyzed the dataset and contributed to writing the manuscript. YT, NH, and KS contributed to writing the manuscript. MM supervised this study. All authors contributed to the article and approved the submitted version.

## Funding

SH had received a Taikichiro Mori Memorial Research Grants, and Research Encouragement Scholarship for Graduate Students of Keio University, the Graduate School of Media and Governance Research Fund, and Keio SFC academic society grants. SH has received the JSPS Research Fellowship for Young Scientists (DC1), The Keio University Doctorate Student Grant-in-Aid Program from Ushioda Memorial Fund. YN has received a Grant-in-Aid for Scientific Research (B) (21H02813) from the Japan Society for the Promotion of Science (JSPS), research grants from Japan Agency for Medical Research and Development (AMED), investigator-initiated clinical study grants from Teijin Pharma Ltd. and Inter Reha Co., Ltd. He has also received research grants from Japan Health Foundation, Meiji Yasuda Mental Health Foundation, Mitsui Life Social Welfare Foundation, Takeda Science Foundation, SENSHIN Medical Research Foundation, Health Science Center Foundation, Mochida Memorial Foundation for Medical and Pharmaceutical Research, Taiju Life Social Welfare Foundation, and Daiichi Sankyo Scholarship Donation Program. He has received speaker’s honoraria from Dainippon Sumitomo Pharma, Mochida Pharmaceutical Co., Ltd., Yoshitomiyakuhin Corporation, Qol Co., Ltd., Teijin Pharma Ltd., Takeda Pharmaceutical Co., Ltd., and Lundbeck Japan Co. Ltd. within the past 5 years outside the submitted work. He also receives equipment-in-kind support for an investigator-initiated study from Magventure Inc., Inter Reha Co., Ltd., Brainbox Ltd., and Miyuki Giken Co., Ltd. SN has received grants from Japan Society for the Promotion of Science (18H02755, 22H03002), Japan Agency for Medical Research and development (AMED), Japan Research Foundation for Clinical Pharmacology, Naito Foundation, Takeda Science Foundation, Watanabe Foundation, Uehara Memorial Foundation, and Daiichi Sankyo Scholarship Donation Program within the past 3 years. He has also received research support, manuscript fees or speaker’s honoraria from Dainippon Sumitomo Pharma, Meiji-Seika Pharma, Otsuka Pharmaceutical, Shionogi, and Yoshitomi Yakuhin within the past 3 years. MM has received speaker’s honoraria from Byer Pharmaceutical, Daiichi Sankyo, Dainippon-Sumitomo Pharma, Eisai, Eli Lilly, Fuji Film RI Pharma, Hisamitsu Pharmaceutical, Janssen Pharmaceutical, Kyowa Pharmaceutical, Mochida Pharmaceutical, MSD, Mylan EPD, Nihon Medi-physics, Nippon Chemipher, Novartis Pharma, Ono Yakuhin, Otsuka Pharmaceutical, Pfizer, Santen Pharmaceutical, Shire Japan, Takeda Yakuhin, Tsumura, and Yoshitomi Yakuhin within the past 3 years. Also, he received grants from Daiichi Sankyo, Eisai, Pfizer, Shionogi, Takeda, Tanabe Mitsubishi, and Tsumura within the past 3 years outside the submitted work. SF has received Grants-in-Aid from JSPS (20H04092 and 21K19734), JST COI-NEXT grant (JPMJPF2203), and research grants from Keio University Academic Development Funds.

## Conflict of interest

The authors declare that the research was conducted in the absence of any commercial or financial relationships that could be construed as a potential conflict of interest.

## Publisher’s note

All claims expressed in this article are solely those of the authors and do not necessarily represent those of their affiliated organizations, or those of the publisher, the editors and the reviewers. Any product that may be evaluated in this article, or claim that may be made by its manufacturer, is not guaranteed or endorsed by the publisher.

## Supplementary material

The Supplementary material for this article can be found online at: https://www.frontiersin.org/articles/10.3389/fnins.2023.1196805/full#supplementary-material

## Footnotes

^1^ https://github.com/CIC-methods/FID-A

^2^ http://www.gabamrs.com
