# Supplementary material for: Glutamatergic neurometabolite levels in the caudate are associated with the ability of rhythm production
Source: Front Neurosci. 2023 Aug 4;17:1196805. doi: 10.3389/fnins.2023.1196805 (PMC10436544; doi:10.3389/fnins.2023.1196805)

Supplementary Table1. Relaxation times and relative water tissue content values

compartment	Water concentration (mmol H20 / kg tissue)	T1 (ms)	T2 (ms)
**CSF**	55556	4000	2000
**GM**	43300	1200	100
**WM**	35880	800	80

Supplementary Table2. Spectrum qualities and tissue heterogeneity values

	Caudate (mean ± SD)	dACC (mean ± SD)
**FWHM (Hz)**	0.063±0.008	0.037±0.005
**SNR**	15.364±2.150	23.852±4.148
**%SD Glx**	8.364±1.965	5.185±0.786
**GM/(GM+WM)**	35.458±5.616	74.237±3.742
**GM ratio**	0.286±0.045	0.532±0.032
**WM ratio**	0.524±0.076	0.186±0.035
**CSF ratio**	0.190±0.076	0.282±0.047

Supplementary Figure 1: The flowchart showing the reasons for exclusion from the analyses

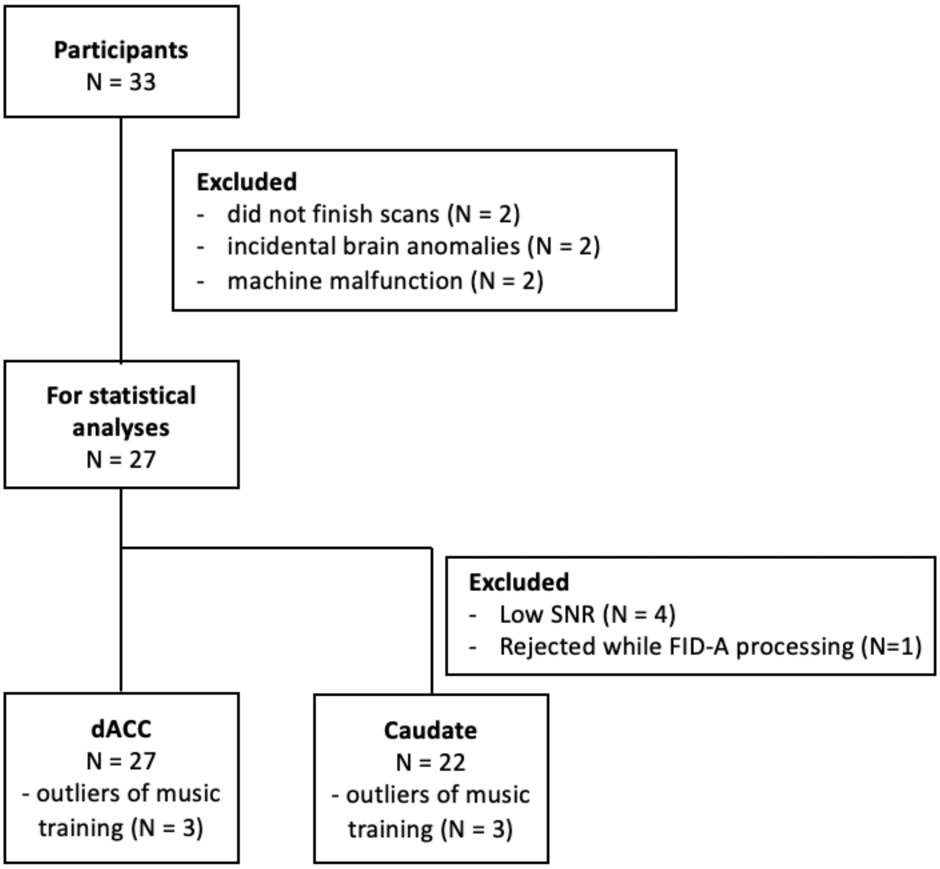

Supplement: Supplementary file 1 [file Data_Sheet_1.pdf]
